# Bisoprolol, Known to Be a Selective β_1_-Receptor Antagonist, Differentially but Directly Suppresses I_K(M)_ and I_K(erg)_ in Pituitary Cells and Hippocampal Neurons

**DOI:** 10.3390/ijms20030657

**Published:** 2019-02-02

**Authors:** Edmund Cheung So, Ning-Ping Foo, Shun Yao Ko, Sheng-Nan Wu

**Affiliations:** 1 Department of Anesthesia, An Nan Hospital, China Medical University, Tainan 70965, Taiwan; edmundsotw@gmail.com (E.C.S.); D23923@mail.tmanh.org.tw (N.-P.F.); 2 Department of Anesthesia, China Medical University, Taichung 40402, Taiwan; 3 Graduate Institute of Medical Sciences, Chang Jung Christian University, Tainan 71101, Taiwan; syko@mail.cjcu.edu.tw; 4 Department of Emergency Medicine, An Nan Hospital, China Medical University, Tainan 70965, Taiwan; 5 Institute of Basic Medical Sciences, National Cheng Kung University Medical College, Tainan 70101, Taiwan; 6 Department of Physiology, National Cheng Kung University Medical College, Tainan 70101, Taiwan

**Keywords:** bisoprolol, M-type K^+^ current, M-type K^+^ channel. erg-mediated K^+^ current, membrane potential, pituitary cell

## Abstract

Bisoprolol (BIS) is a selective antagonist of β_1_ adrenergic receptors. We examined the effects of BIS on M-type K^+^ currents (I_K(M)_) or *erg*-mediated K^+^ currents (I_K(erg)_) in pituitary GH_3,_ R1220 cells, and hippocampal mHippoE-14 cells. As GH_3_ cells were exposed to BIS, amplitude of I_K(M)_ was suppressed with an IC_50_ value of 1.21 μM. The BIS-induced suppression of I_K(M)_ amplitude was not affected by addition of isoproterenol or ractopamine, but attenuated by flupirtine or ivabradine. In cell-attached current, BIS decreased the open probability of M-type K^+^ (K_M_) channels, along with decreased mean opening time of the channel. BIS decreased I_K(erg)_ amplitude with an IC_50_ value of 6.42 μM. Further addition of PD-118057 attenuated BIS-mediated inhibition of I_K(erg)_. Under current-clamp conditions, BIS depolarization increased the firing of spontaneous action potentials in GH_3_ cells; addition of flupirtine, but not ractopamine, reversed BIS-induced firing rate. In R1220 cells, BIS suppressed I_K(M)_; subsequent application of ML-213(Kv7.2 channel activator) reversed BIS-induced suppression of the current. In hippocampal mHippoE-14 neurons, BIS inhibited I_K(M)_ to a greater extent compared to its depressant effect on I_K(erg)_. This demonstrated that in pituitary cells and hippocampal neurons the presence of BIS is capable of directly and differentially suppressing I_K(M)_ and I_K(erg)_, despite its antagonism of β_1_-adrenergic receptors.

## 1. Introduction

Bisoprolol (zebeta^®^; BIS), a phenoxy-2-propanol derivative, is recognized as an oral synthetic selective blocker of β_1_-adrenoceptor with antioxidant activity which exerts a number of potentially beneficial pharmacological effects such as atrial fibrillation, heart failure and postural tachycardia syndrome [1,2,3,4]. Due to its lipophilic nature, it facilitates entry into brain tissue to produce regulatory actions on central neurons [5,6]. Previous reports have demonstrated that BIS could bind to β_1_-adrenergic receptors inherently existing in brain areas including the pituitary gland and hippocampus [7,8,9,10,11]. An earlier study has also reported the ability of BIS to elevate blood prolactin level [12]. 

Carvedilol or other blockers of β-adrenergic receptors were indeed previously shown to suppress the amplitude of delayed-rectifier K^+^ currents (*I*_K(DR)_) and of HERG-mediated K^+^ currents [13,14]. There is also evidence showing that BIS appears to modulate a variety of ion currents. For example, previous reports have shown that BIS was able to reverse the down-regulation in mRNA expression of Na^+^, hyperpolarization-activated cation (HCN) and small-conductance Ca^2+^-activated K^+^ (SK) channels in failing hearts [15,16]. Surprisingly, little information is available regarding the underlying mechanism of actions of BIS or other structurally similar compounds on ionic currents in endocrine cells, neuroendocrine cells, or neurons, despite their wide clinical usage [2].

The KCNQ2, KCNQ3, and KCNQ5 genes are known to encode the core subunits of K_V_7.2, K_V_7.3 and K_V_7.5 channels, respectively. The increased activity of these K^+^ channels can generate the M-type K^+^ current (*I*_K(M)_) which is characterized by the activation in response to low threshold voltage and, once activated, displays a slowly activating and deactivating property [17,18,19,20]. Targeting *I*_K(M)_ is growingly recognized as an adjunctive regimen for the treatment of many neurological disorders associated with neuronal hyper-excitability, such as cognitive dysfunction, neuropathic pain and epilepsy [21,22,23,24]. 

The *erg*-mediated K^+^ current (*I*_K(erg)_) is encoded by three different subfamily of genes KCNH giving rise to the pore-forming α-subunit of *erg* (or K_V_11) channels. This type of current is characterized by slow activation and fast inactivation kinetics. Because of its rapid inactivation kinetics, it can result in an inward rectification. The magnitude of these currents in endocrine or neuroendocrine cells can contribute to the maintenance of the resting potential, thereby influencing the discharge frequency of action potentials and the stimulus-secretion coupling in these cells (APs) [25,26,27,28]. Earlier reports in our laboratory have shown the ability of ranolazine, risperidone or methadone to suppress *I*_K(erg)_ in pituitary GH_3_ cells [25,27,29] However, to what extent the presence of BIS exerts any actions on these types of ionic currents (e.g., *I*_K(M)_ and *I*_K(erg)_) remains elusive.

In this study, we wanted to determine the possible underlying mechanism of BIS actions on perturbation of different ionic currents (e.g., *I*_K(M)_, *I*_K(erg)_ and *I*_K(DR)_) in pituitary GH_3_ cells and hippocampal mHippoE-14 neurons. Findings from this present study highlight the evidence to show that BIS can directly and differentially inhibit *I*_K(M)_ and *I*_K(erg)_ in a concentration-dependent manner in pituitary cells and hippocampal neurons. Such suppression of ion currents appears to be direct and is highly unlikely to be linked to its antagonistic action on β_1_ adrenergic receptors.

## 2. Results

### 2.1. Expression of KCNC1 (KV3.1), KCNQ2 (KV7.2) and KCNQ3 (KV7.3) in Pituitary GH_3_ Cells

**Figure 1:**
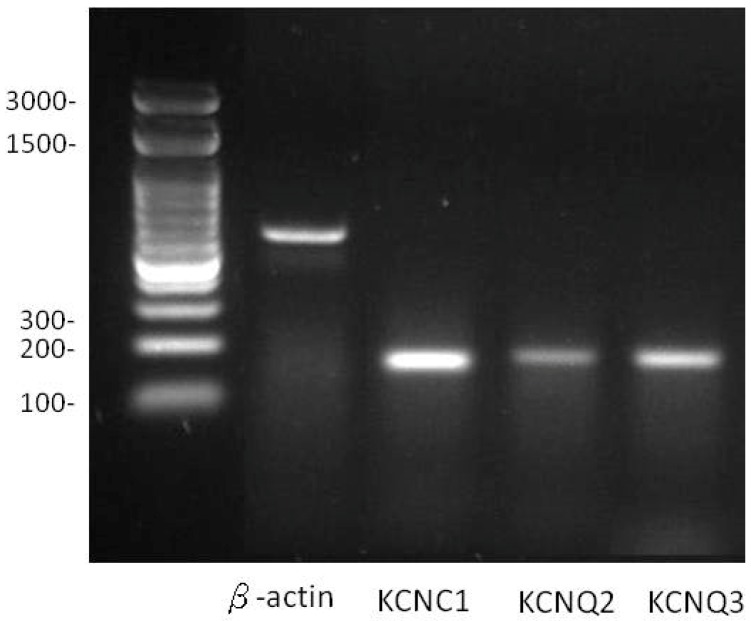
The expression levels of the β-actin, KCNC1 (K_V_3.1), KCNQ2 (K_V_7.2) and KCNQ3 (K_V_7.3) isolated from GH_3_ cells. Total RNA was isolated and RT-PCR analysis was made. Amplified RT-PCR products were obtained using for a marker lane of DNA molecular size (leftmost) and KCNQ2/3 subunit (198 bp). The labelling in the left side indicates the molecular weight.

In pituitary GH_3_ cells, as the M-type K^+^ current (*I*_K(M)_) was to be carried by the product of KCNQ2/KCNQ3 (or K_V_7.2/K_V_7.3) genes, we examined the mRNA levels of these genes. Our RT-PCR analysis demonstrated the mRNA expression of KCNQ2/KCNQ3 in these cells (Figure 1). 

### 2.2. Effect of Bisoprolol (BIS) on I_K(M)_ in Pituitary GH_3_ Cells

**Figure 2:**
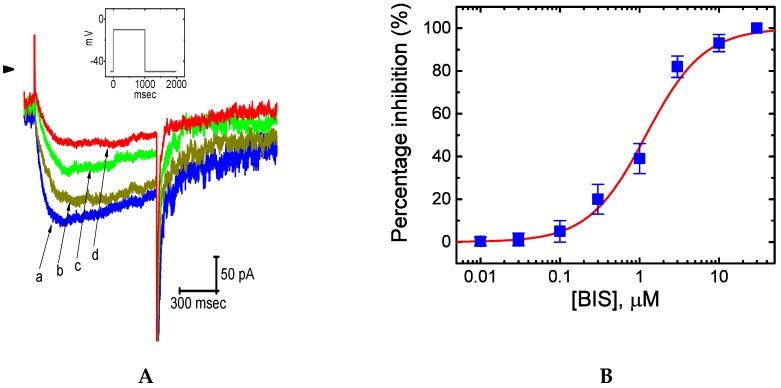

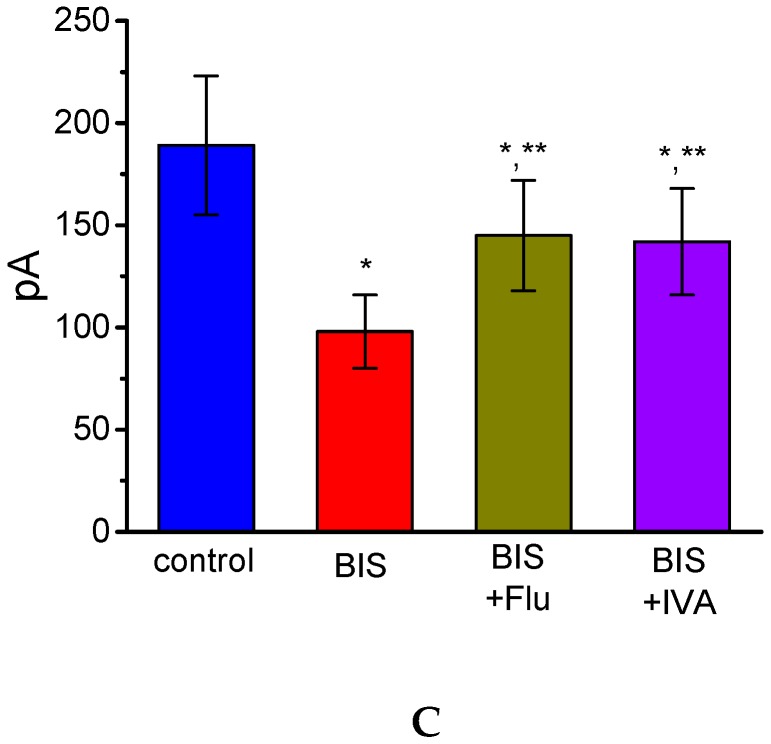
Effect of bisoprolol (BIS) in the amplitude of M-type K^+^ current (*I*_K(M)_) in GH_3_ cells. Cells were bathed in high-K^+^ solution and the pipette was filled with K^+^-containing solution, the composition of which is detailed in Materials and Methods. As the whole-cell mode was established, the depolarizing pulse from −50 to −10 mV (indicated in the Inset of [A)]) was delivered to the examined cells. (**A**) Original *I*_K(M)_ traces in response to long-lasting membrane depolarization. a: control; b: 0.1-μM BIS; c: 0.3-μM BIS; and d: 1-μM BIS. (**B**) Concentration-response curve for BIS-induced inhibition of *I*_K(M)_ in GH_3_ cells. The amplitude of *I*_K(M)_ during cell exposure to different BIS concentrations (0.01–30 μM) was compared with the control value (mean ± SEM; *n* = 12 for each data point). The sigmoidal smooth line represents a best fit to a modified Hill function described in Materials and Methods. The values for IC_50_, maximally inhibited percentage of *I*_K(M)_, and the Hill coefficient were 1.21μM, 100 %, and 1.2, respectively. (**C**) Summary bar graph showing the effects of BIS, BIS plus flupirtine, and BIS plus ivabradine on *I*_K(M)_ amplitude (mean ± SEM; *n* = 9 for each bar). BIS: 1-μM BIS; Flu: 10 μM flupirtine; IVA: 10 μM ivabradine. * Significantly different from control (*p* < 0.05) and ** significantly different from BIS (1 μM) alone group (*p* < 0.05).

In the first set of whole-cell current recordings, we bathed the cells in high-K^+^, Ca^2+^-free Tyrode’s solution, the composition of which is described above, and the recording pipette was filled with K^+^-containing solution. Once the whole-cell model was established, the examined cell was maintained at −50 mV and the depolarizing step to −10 mV with a duration of 1 sec was delivered. As shown in Figure 2, upon long-lasting membrane depolarization to −10 mV, K^+^ inward current was readily evoked, which was responsive to low threshold voltage and displayed both the slowly activating and deactivating properties. This type of K^+^ currents in response to depolarizing step was sensitive to inhibition by linopirdine (10 μM) or pioglitazone (10 μM), and it was hence identified as an *I*_K(M)_ [17,18,19,20]. Of particular interest is how as GH_3_ cells were exposed to BIS, the *I*_K(M)_ amplitude in response to such depolarizing step was progressively diminished in a concentration-dependent manner (Figure 2A,B). For example, addition of 1-μM BIS significantly decreased current amplitude from 196 ± 14 to 97 ± 11 pA (*n* = 12, *p* < 0.05). After washout of the agent, current amplitude returned to 191 ± 14 pA *(n* = 11, *p* < 0.05). Concomitant with this, the value of activation time constant (τ_act_) during the exposure to 1-μM BIS was noted to increase significantly from 102 ± 9 to 182 ± 11 msec *(n* = 12, *p* < 0.05) and that of deactivating time constant (τ_deact_) was decreased from 168 ± 11 to 66 ± 8 msec *(n* = 12, *p* < 0.05). 

The relationship between the BIS concentration (0.01–30 μM) and the *I*_K(M)_ amplitude was further constructed and is plotted in Figure 2B. Fitting the concentration-response curve with the modified Hill equation as described in Materials and Methods for details yielded a half-maximal concentration (i.e., IC_50_) of 1.21 μM and a slope coefficient of 1.2. BIS at a concentration of 30 μM nearly abolished current amplitude. Additionally, in the continued presence of 1-μM BIS, further addition of either 10 μM flupirtine or 10 μM ivabradine was noted to attenuate its suppression of *I*_K(M)_ (Figure 2C). Flupirtine was reported to stimulate *I*_K(M)_ [30], while ivabradine known to suppress hyperpolarization-activated cation current might increase *I*_K(M)_ amplitude in these cells. Conversely, subsequent application of neither isoproterenol (1 μM) nor ractopamine (10 μM) had any perturbations on BIS-induced block of *I*_K(M)_. Isoproterenol or ractopamine (a synthetic phenoethanolamine salt) was previously demonstrated to stimulate β-adrenergic receptors [31,32,33,34,35]. However, addition of neither isoproterenol (1 µM) nor ractopamine (10 µM) alone produced any effects on *I*_K(M)_. The results led us to suggest that BIS has a significant depressant action on *I*_K(M)_ functionally expressed in GH_3_ cells, which appears to be unlinked to binding to β-adrenergic receptors. 

### 2.3. Inhibitory Effect of BIS on I_K(M)_ Elicited by High Frequency Depolarizing Pulse

**Figure 3:**
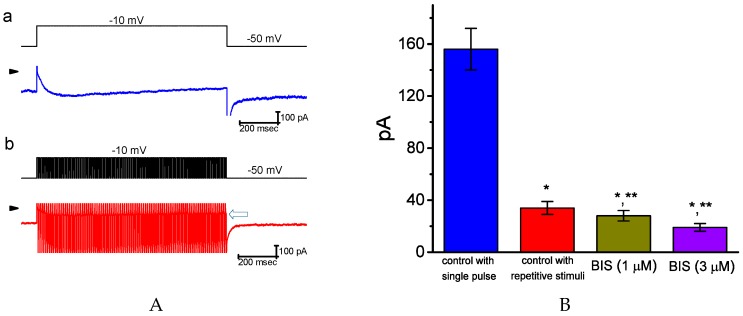
Accumulative activation of *I*_K(M)_ during repetitive stimuli obtained in the absence and presence of BIS in GH_3_ cells. Cells were bathed in high-K^+^, Ca^2+^-free solution. (**A**) Representative current trace in response to single step or repetitive pulses (b). In the lower part of (a), current was evoked in response to single membrane depolarization from−50 to −10 mV with a duration of 1 sec (indicated in the upper part of (a)), whereas that in (b) was obtained during rapid repetitive depolarizations to −10 mV (indicated in the lower part of (b)). The individual depolarizing pulse used to elicit inward K^+^ current lasted 6 msec with a total duration of 1 sec. Arrowheads in (a) and (b) indicate the zero current level, while open arrow indicated in (b) depicts the level of *I*_K(M)_ elicited. The activation time constants for control *I*_K(M)_ traces in response to single step and repetitive depolarizing pulses were 53 and 69 msec, respectively. (**B**) Summary bar graph showing the effects of BIS (1 and 3 μM) on the amplitude of *I*_K(M)_ during repetitive depolarizations (mean ± SEM; *n* = 13 for each bar). Current amplitude was measured at the end of single step or repetitive depolarizing pulses (i.e., 1 sec). *or** indicates significantly different from the controls taken with single depolarizing step *(p* < 0.05) or repetitive stimuli *(p* < 0.05), respectively.

Some neurons, neuroendocrine cells, or endocrine cells can display firing behavior with high frequency [25,36,37]. For this reason, in another set of experiments, we sought to evaluate whether BIS affects the magnitude of *I*_K(M)_ elicited by brief repetitive depolarizations. Under control conditions, a single 1-sec depolarizing step to −10 mV from a holding potential of −50 mV evoked *I*_K(M)_ amplitude of 156 ± 16 pA (*n* = 13) with the τ_act_ value of 53 ± 7 msec (*n* = 13). However, the *I*_K(M)_ amplitude for 1-sec repetitive pulses to −10 mV, each of which lasted 6 msec with 4-msec intervals at the level of −50 mV between each abrupt pulse was evidently reduced to 34 ± 5 pA (*n* = 13), together with a significant prolongation of τ_act_ value to 69 ± 8 msec (*n* = 13). A representative example of *I*_K(M)_ traces in response to either single pulse or repetitive pulses is illustrated in Figure 3A. As shown in Figure 3B, addition of BIS was able to suppress the *I*_K(M)_ amplitude elicited by high-frequency depolarizing pulse. For example, as cells were exposed to 3-μM BIS, current amplitude measured at the end of a train of short repetitive stimuli was further reduced by 56 ± 2 % to 19 ± 3 pA (*n* = 13, *p* < 0.05). Therefore, *I*_K(M)_ activation was reduced by repetitive stimuli, and the inhibition by this agent of *I*_K(M)_ amplitude still remained effective. However, the magnitude of its block appears to become smaller, as compared to those with a single long depolarizing pulse.

### 2.4. Effect of BIS on Deactivating I_K(M)_ Elicited Upon Return to Membrane Hyperpolarization with Varying Duration

**Figure 4:**
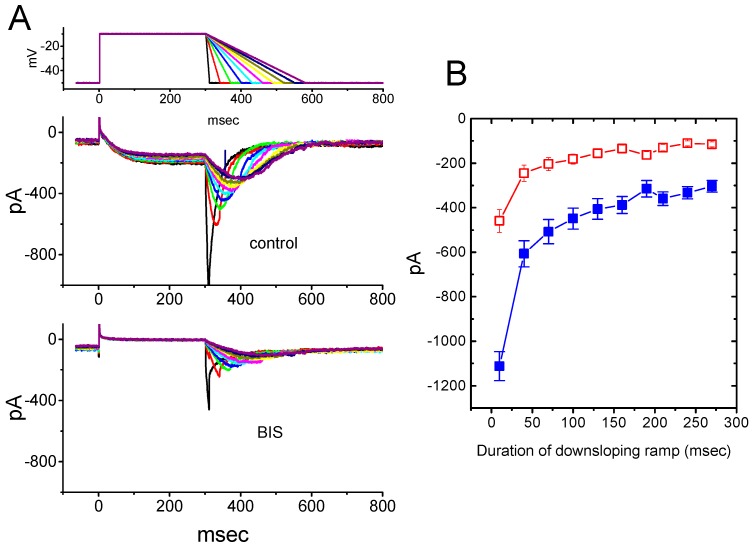
Effect of BIS on the deactivating *I*_K(M)_ in response to depolarizing pulse with different durations (10-280 msec) of falling phase which was used to mimic different repolarizing slope of bursting pattern. (**A**) Superimposed current traces in response to the uppermost voltage protocol obtained in the absence (upper) and presence (lower) of 10-μM BIS. The uppermost part indicates the voltage profile applied. (**B**) Effect of BIS on deactivating *I*_K(M)_ upon return to −50 mV with different durations (mean ± SEM; *n* = 11 for each data point). The peak amplitude of deactivating *I*_K(M)_ was taken at different durations of falling phase. ■: control; □: in the presence of 3-μM BIS.

Previous studies have shown that the magnitude of *I*_K(M)_ might influence the falling phase of bursting firing or spike after-depolarizations [18,38]. Thus, we wanted to determine how BIS exerted any perturbations on deactivating *I*_K(M)_ in response to the down sloping ramp pulse from −10 to −50 mV with varying durations. As shown in Figure 4A and 4B, upon return to −50 mV, as the slope of ramp pulse was slowed, the peak amplitude of deactivating *I*_K(M)_ became exponentially decreased with a time constant of 98 ± 8 msec (*n* = 11). However, as cells were exposed to 3-μM BIS, the peak amplitude of the current was significantly and exponentially decreased, with a time constant of 65 ± 7 msec (*n* = 11). For example, as the duration of downslope ramp was set at 40 msec (i.e., slope = −1 V/sec), the addition of 3-μM BIS decreased peak amplitude by 59 ± 2 % from 607 ± 50 to 245 ± 36 pA (*n* = 11, *p* < 0.05). The results thus indicated that, as the duration of down sloping ramp pulse increased, the amplitude of deactivating *I*_K(M)_ would be exponentially decreased, and that the presence of BIS decreased current magnitude in a time-dependent fashion.

### 2.5. Inhibitory Effect of BIS on the Activity of Single M-type K^+^ (K_M_) Channels in GH_3_ Cells

**Figure 5:**
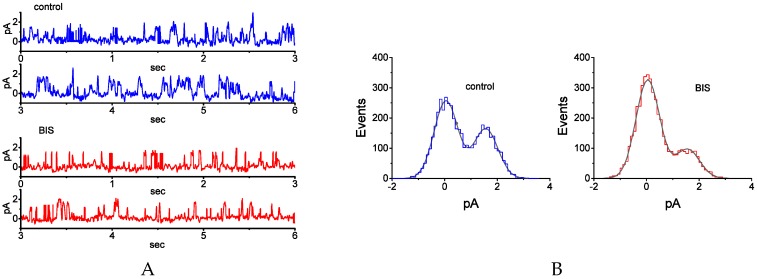
Effect of BIS on single channel activity of M-type K^+^ (K_M_) channels in GH_3_ cells. In these current recordings, cells were bathed in high-K^+^, Ca^2+^-free solution and the recording pipette was filled with low-K^+^ (5.4 mM) solution, the composition of which is described in Materials and Methods. (**A**) Original single K_M_ channels obtained in the absence (upper) and presence of 1-μM BIS (lower). The potential was maintained at 0 mV relative to the bath. The upward deflection indicates the opening event of the channel. (**B**) Amplitude histogram obtained in the control (left) and during the exposure to 1-μM BIS (right). The smooth line shown in each histogram indicates the Gaussian curve.

The cell-attached configuration of the patch-clamp technique was used to investigate the effects of BIS on the activity of single K_M_ channels expressed in these cells. In these experiments, we bathed cells in high-K^+^, Ca^2+^-free solution and the recording pipette was filled with low-K^+^ (5.4 mM) solution. As shown in Figure 5A, the activity of K_M_ channels, which occurred in rapid open-closed transitions, was clearly detected, as the membrane was maintained at 0 mV relative to the bath. The addition of 1-μM BIS significantly decreased channel open probability from 0.129 ± 0.006 to 0.056 ± 0.003 (*n* = 12, *p* < 0.01). After washout of the drug, channel activity was returned to 0.114 ± 0.004 (*n* = 10, *p* < 0.01). Based on an amplitude histogram (Figure 5B), the single-channel amplitude did not differ between the absence and presence of 1-μM BIS. Moreover, in continued presence of 1 µM BIS, further addition of neither diazoxide (10 μM), 9-phenenthrol (10 μM), nor GMQ (10 μM) attenuated its suppression of channel activity. Diazoxide and 9-phenanthrol are the openers of ATP-sensitive K^+^ and intermediate-conductance Ca^2+^-activated K^+^ channels [39], respectively, while GMQ can activate large-conductance Ca^2+^-activated K^+^ channels [40]. These results strongly indicate that the channel activity suppressed by the presence of BIS ascribes primarily from K_M_ channels.

### 2.6. Effect of BIS on Mean Open Time of K_M_ Channels in GH_3_ Cells

**Figure 6:**
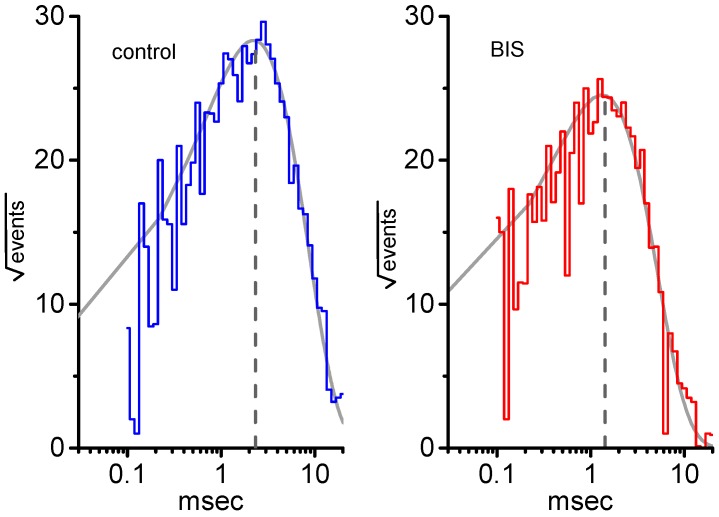
Effect of BIS on mean open time of K_M_ channels recorded from GH_3_ cells. In control, data were obtained from measurements of 480 channel openings, with a total recording time of 2 min, whereas in the presence of 1-μM BIS, data were from 371 channel openings, with a total recording time of 3 min. Note that the ordinate and abscissa indicate the square root of the event number (i.e., $\sqrt{\mathrm{event}}$) and the logarithm of open time (msec) and, respectively. The smooth line shown in each lifetime distribution was least-squares fitted using a single-exponential function, and the vertical broken line illustrates the value of the time constant.

After observing that during the exposure to BIS, the open-time duration of K_M_ channels in GH_3_ tended to be shortened with no change in single-channel amplitude (Figure 5A), we further examined and analyzed the kinetic properties of K_M_ channels obtained with or without addition of BIS. As depicted in Figure 6, the distribution of open durations was least-squares fitted by a mono-exponential function. During the exposure to 1-μM BIS, the mean open time of these channels was significantly reduced to 1.67 ± 0.05 msec from the control length of 2.32 ± 0.09 msec (*n* = 12, *p* < 0.05). Therefore, BIS-induced changes in K_M_-channel activity may have been a result of the decrease in the duration of the open state, rather than of any modification in single-channel conductance.

### 2.7. Lack of BIS Effect on Single-Channel Conductance of K_M_ Channels

**Figure 7:**
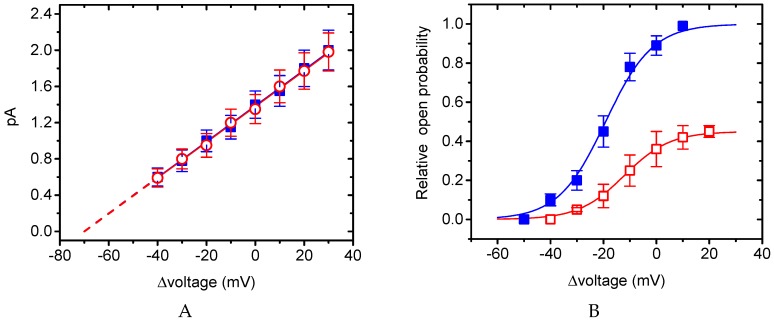
Effect of BIS on *I-V* relationship and activation curve of K_M_ channels in GH_3_ cells. (**A**) Averaged *I-V* relationships of single K_M_-channel currents in the absence (■) and presence (○) of 1-μM BIS (mean ± SEM; *n* = 9 for each data point). The broken line is pointed toward the value of the reversal potential with -70 mV. (**B**) Activation curves of K_M_ channels in the absence (■) and presence (□) of 1-μM BIS (mean ± SEM; *n* = 9 for each data point). Channel activity measured under cell-attached current recordings was measured at different potentials relative to the bath. The smooth curves overlaid onto each data set were least-squares fitted with the Boltzmann equation (see Materials and Methods for details).

We next measured the amplitude and open probability of single K_M_ channels at a series of voltages ranging between −40 and +30 mV. Throughout the voltage range examined, averaged *I-V* relationships of K_M_ channels with or without BIS (1 μM) addition were analyzed and then compared (Figure 7A). In the control, fitting these single-channel amplitudes with a linear regression revealed K_M_ channels of 19.8 ± 0.4 pS (*n* = 9). The value for these channels did not differ significantly from that (19.7 ± 0.4 pS, *n* = 9, *p* > 0.05) during exposure to 1-μM BIS. It is clear from the results that addition of this compound is unable to modify single-channel conductance, although it can significantly exert a depressant on the probability of K_M_ channels that would be open.

### 2.8. Activation Curve of K_M_ Channels Modified by the Presence of BIS

Figure 7B shows the activation curve of K_M_ channels taken with or without addition of 1-μM BIS. In these experiments, channel open probabilities were measured at different voltages relative to the bath. The plot of channel open probability of K_M_ channels as a function of Δvoltage was derived and then least-squares fitted with the Boltzmann function as described in Materials and Methods for details. In control (i.e., in the absence of BIS), P_max_ = 0.99 ± 0.01, V_1/2_ = −19.2 ± 1.2 mV, q = 2.83 ± 0.05 *e* (*n* = 9), whereas in the presence of 1-μM BIS, P_max_ = 0.45 ± 0.01, V_1/2_ = −12.1 ± 0.9 mV, q = 2.85 ± 0.05 *e* (*n* = 9). Therefore, it is clear from these results that the presence of BIS not only produced a decrease in the maximal open probability of these channels, but also significantly shifted the activation curve along the voltage axis to depolarized potential by approximately 7 mV. In contrast, minimal change on the gating charge of such activation curve was demonstrated in the presence of BIS. Consequently, as cells were exposed to 1-μM BIS, the difference in free energy (ΔG = -q × F × V_1/2_, where F = 23,063 cal × V^−1^ × *e*^−1^) required for activation of K_M_ channels was significantly reduced from 1.25 ± 0.04 to 0.79 ± 0.04 Kcal (*n* = 9, *p* < 0.05).

### 2.9. Effect of BIS on Erg-Mediated K^+^ Current (I_K(erg)_) in GH_3_ Cells

**Figure 8:**
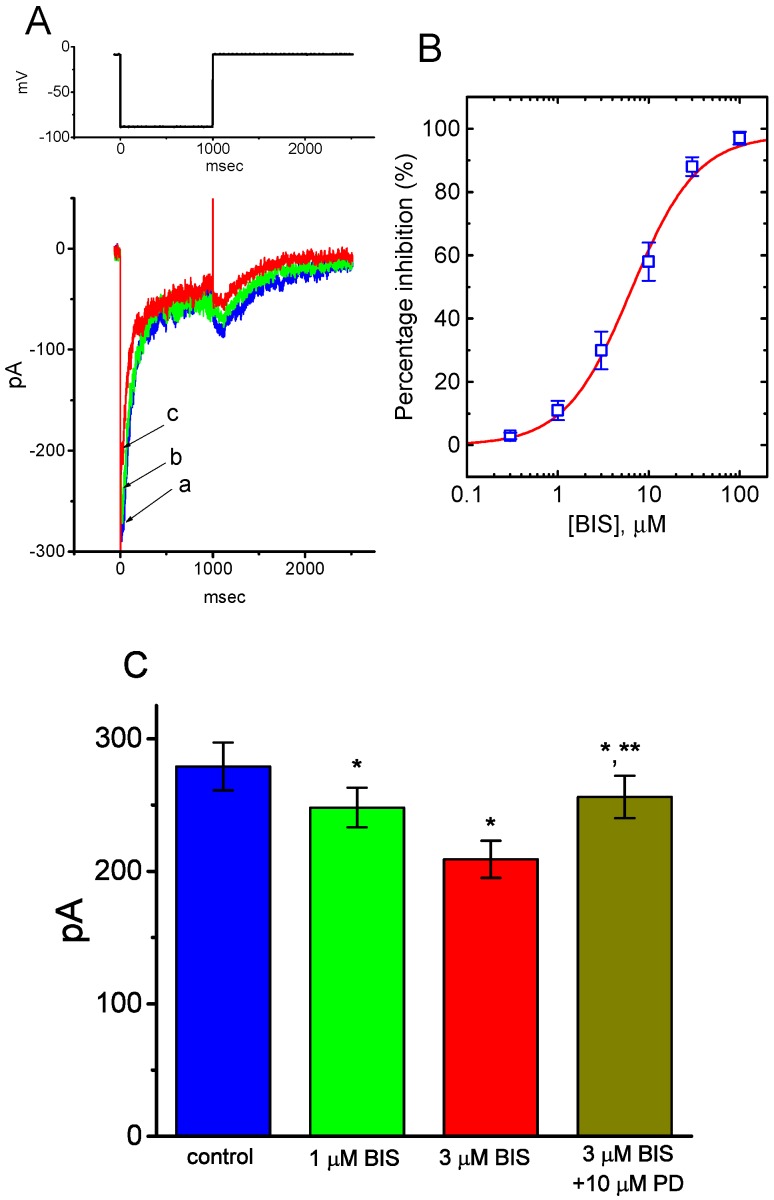
Inhibitory effect of BIS on *erg*-mediated K^+^ currents (*I*_K(erg)_) in GH_3_ cells. In these experiments, we bathed the cells in high-K^+^, Ca^2+^-free solution and the pipette used was filled with K^+^-containing solution. (**A**) Original current traces in response to membrane hyperpolarization from −10 mV (indicated in the upper part). a: control; b: 1-μM BIS; c: 3-μM BIS. (**B**) Percentage inhibition of *I*_K(erg)_ produced by different concentrations (0.3–100 μM) of BIS. Each cell was hyperpolarized from −10 to -80 mV, and current amplitudes at the end of hyperpolarizing step with or without BIS were measured and compared (mean ± SEM; *n* = 8 for each data point). Smooth line represents the best fit to a modified Hill function (see Materials and Methods for details). (**C**) Summary bar graph showing the effect of BIS and BIS plus PD-118057 on *I*_K(erg)_ amplitude (mean ± SEM; *n* = 9 for each bar). *I*_K(erg)_ elicited by hyperpolarization from −10 to −80 mV was measured at the beginning of each hyperpolarizing pulse. PD: PD-118057 (10 μM). * Significantly different from control (*p* < 0.05) and ** significantly different from 3-μM BIS alone group (*p* < 0.05).

Previous studies have reported the importance of *I*_K(erg)_ functionally expressed in pituitary cells including GH_3_ cells [41,42]. Some of β-adrenoceptor blockers have been previously demonstrated to suppress HERG K^+^ or other types of voltage-gated K^+^ channels [13,14]. As such, we further studied the possible perturbation by BIS on this type of K^+^ currents (i.e., *I*_K(erg)_). Interestingly, as cells were exposed to different concentrations of BIS, the amplitude of *I*_K(erg)_ elicited by membrane hyperpolarization progressively decreased in a concentration-dependent manner (Figure 8A,B). From nonlinear least-squares fit to the data points, the IC_50_ value needed to exert its inhibitory effect on the amplitude of deactivating *I*_K(erg)_ was calculated to be 6.42 μM with a Hill coefficient of 1.2, and the agent at a concentration of 100 μM could nearly abolish current amplitude. Moreover, during continued presence of 3-μM BIS, subsequent addition of 10-μM PD-118057 significantly attenuated the decrease of *I*_K(erg)_ produced by this agent. PD-118057 was previously reported to activate *I*_K(erg)_ in heart cells [43]. Therefore, BIS could effectively suppress *I*_K(erg)_ amplitude, despite its inhibitory effect on *I*_K(erg)_ to a lesser extent as compared with that on *I*_K(M)_ described above. Similar to previous observations [14] showing the ability of propranolol and its derivative to block HERG channels, BIS-induced inhibition of *I*_K(erg)_ tends to be independent of its agonistic effect on β-adrenergic receptors.

### 2.10. The Inhibition of Delayed-Rectifier K^+^ Current (I_K(DR)_) in GH_3_ cells Caused by the Presence of BIS

**Figure 9:**
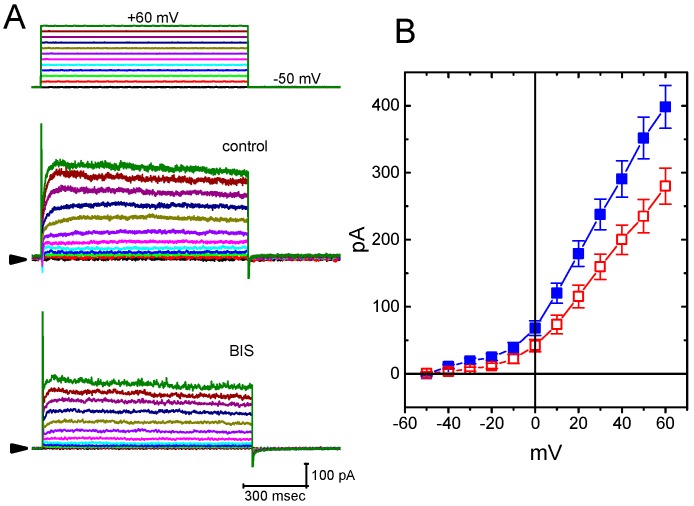
Effect of BIS on delayed-rectifier K^+^ current (*I*_K(DR)_) in GH_3_ cells. In these whole-cell current recordings, cells were bathed in Ca^2+^-free Tyrode’s solution. The examined cells were held at −50 mV and a series of voltage step ranging between −50 and +60 mV. (**A**) Superimposed current traces obtained in the absence (upper) and presence (lower) of 30-μM BIS. The uppermost part indicates the voltage protocol used and arrowhead is the zero current level. (**B**) Averaged *I-V* relationships of *I*_K(DR)_ obtained in the absence (■) and presence (○) of 30-μM BIS (mean ± SEM; *n* = 11 for each data point). Current amplitude was measured at the end of each depolarizing step from a holding potential of −50 mV.

An earlier report showed the ability of carvedilol to suppress *I*_K(DR)_ in NG108-15 neuronal cells [13]. We also explored whether or not the presence of BIS produced any effects on another types of K^+^ currents (e.g., *I*_K(DR)_) in these cells. In these experiments, we bathed cells in Ca^2+^-free Tyrode’s solution, and, once whole-cell mode was firmly established, the examined cells were maintained at −50 mV and a series of depolarizing voltage steps ranging between −50 and +60 mV was then delivered. BIS at a concentration of 10 μM was not found to have significant effect on *I*_K(DR)_ elicited throughout the entire voltage-clamp steps applied (Figure 9). However, upon cell exposure to BIS (30 μM), the *I*_K(DR)_ amplitude was significantly decreased. For example, at the level of +50 mV, current amplitude decreased from 352 ± 31 to 235 ± 25 pA (*n* = 11, *p* < 0.05). Therefore, distinct from *I*_K(M)_ or *I*_K(erg)_, the *I*_K(DR)_ is relatively resistant to suppression by BIS.

### 2.11. Effect of BIS on Spontaneous Action Potentials (APs) in GH_3_ Cells

**Figure 10:**
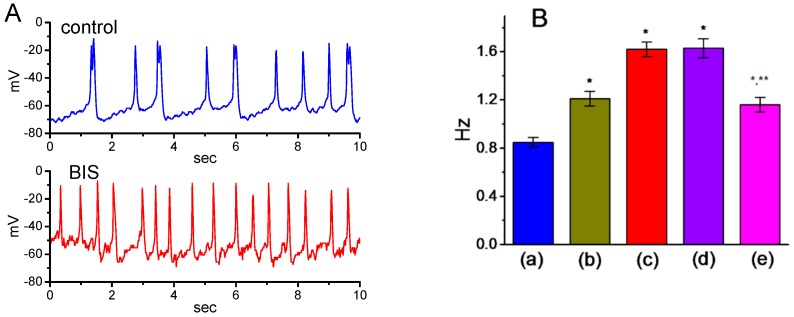
Effects of BIS on spontaneous action potentials (APs) in GH_3_ cells. In these current-clamp voltage recordings, cells were bathed in normal Tyrode’s solution containing 1.8 mM CaCl_2_, and the recording pipette was filled with K^+^-containing solution. (**A**) Original potential trace obtained in the absence (upper) and presence (lower) of 3-μM BIS. (**B**) Summary bar graph showing the effects of BIS, BIS plus ractopamine, and BIS plus flupirtine on the firing frequency of spontaneous APs in GH_3_ cells (mean ± SEM; *n* = 8 for each bar). a: control; b: 1-μM BIS c: 3-μM BIS; d: 3-μM BIS plus 10 μM ractopamine; e: 3-μM BIS plus 10 μM flupirtine. * Significantly different from control (*p* < 0.05) and ** significantly different from the 3-μM BIS alone group (*p* < 0.05).

In a separate set of experiments, we explored whether the presence of BIS perturbs any changes in membrane potential recorded from these cells. As shown in Figure 10A, as the current-clamp voltage recordings were established, spontaneous APs with a firing frequency of 0.85 ± 0.06 Hz (*n* = 8) were readily observed. As cells were exposed to BIS, membrane became depolarized and the firing frequency was concomitantly raised, as evidenced by a significant raise in the firing frequency to 1.62 ± 0.06 Hz (*n* = 8, *p* < 0.05) during the exposure to 3-μM BIS. In continued presence of 3-μM BIS, subsequent addition of 10 μM flupirtine significantly attenuated BIS-induced increase in the firing of spontaneous APs to 1.16 ± 0.06 Hz (*n* = 8, *p* < 0.05); however, that of 10 μM ractopamine did not have any effects on it (Figure 10B). It is therefore conceivable that the observed effect of BIS on the firing patterns of GH_3_ cells appears to be independent of binding to β-adrenergic receptors and could be partly explained by its suppression of *I*_K(M)_.

### 2.12. Suppressive Effect of BIS on I_K(M)_ in Pituitary R1220 Cells

**Figure 11:**
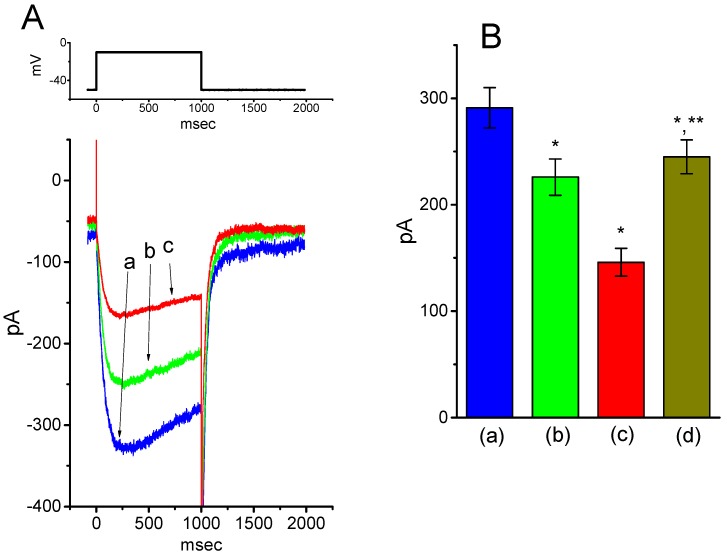
Inhibitory effect of BIS on *I*_K(M)_ recorded from rat pituitary R1220 cells. In this set of recordings, similar experimental conditions to that in GH_3_ cells described above were applied. (**A**) Original *I*_K(M)_ elicited by membrane depolarization from −50 to −10 mV. a: control; b: 0.3-µM BIS and c: 1 µM BIS. (**B**) Summary bar graph showing effect of BIS and BIS plus flupirtine on *I*_K(M)_ in these cells (mean ± SEM; *n* = 12 for each bar). a: control; b: 0.3-µM BIS; c: 1-µM BIS; d: 1-µM BIS plus 10-µM ML-213. * Significantly different from control (*p* < 0.05) and ** significantly different from 1-µM BIS alone group (*p* < 0.05).

We next wanted to test if *I*_K(M)_ in another type of pituitary cells (e.g., pituitary R1220 cells) can be influenced by BIS. This type of rat pituitary cells, originally derived from ScienCell (Carlsbad, CA, USA), was previously reported to express β_1_-adrenergic receptors [8]. As depicted in Figure 11, as cells were exposed to different BIS concentrations, the amplitude of *I*_K(M)_ in response to depolarizing step was progressively decreased, in combination with a slowing in activation time course of *I*_K(M)_. Moreover, with the presence of 1-μM BIS, further addition of ML-213 (10 μM), an activator of *I*_K(M)_ [44], was able to attenuate BIS-induced inhibition of *I*_K(M)_, despite inability of further isoproterenol addition to exert any effect on such block. These results thus appear to be indistinguishable from those described above in GH_3_ cells. 

### 2.13. Effect of BIS on I_K(M)_ Recorded from Hippocampal mHippoE-14 Cells

**Figure 12:**
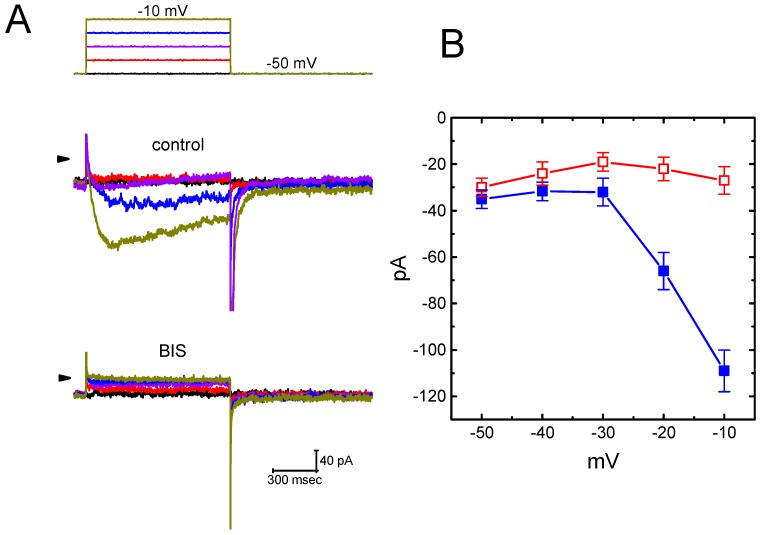
Effect of BIS on *I*_K(M)_ in hippocampal mHippoE-14 cells. In these experiments, we bathed the cells in high-K^+^, Ca^2+^-free solution, and the examined cell was depolarized from −50 mV to various voltages ranging between −50 and −10 mV. (**A**) Original current traces obtained in the absence (upper) and presence (lower) of 3 µM BIS. The uppermost part indicates the voltage protocol applied and arrowhead is the zero current level. (**B**) Averaged *I-V* relationship of *I*_K(M)_ (mean ± SEM; *n* = 9 for each data point). ■: control; □: 3 µM BIS. Current amplitude was measured at the end of each voltage step.

In a final set of experiments, we wanted to determine whether or not *I*_K(M)_ inherently in central neurons (e.g., hippocampal mHippoE-14 cells) [21] is subject to suppression by this compound. The experimental profiles used were similar to those for pituitary cells. As depicted in Figure 12A,B, the *I*_K(M)_ elicited by different depolarizing steps was suppressed by the presence of BIS. For example, at the level of −10 mV, the exposure to 3-μM BIS caused a significant reduction of *I*_K(M)_ amplitude from 109 ± 9 pA to 27 ± 6 pA (*n* = 9, *p* < 0.05). After washout of BIS, current amplitude returned to 98 ± 9 pA (*n* = 9, *p* < 0.05). The averaged *I-V* relationships of *I*_K(M)_ obtained with or without BIS addition are illustrated in Figure 12B. However, isoproterenol (1 µM) had little or no effects on *I*_K(M)_ amplitude (108 ± 9 pA [control] versus 107 ± 10 pA [in the presence of 1 µM isoproterenol], *n* = 9, *p* > 0.05). Therefore, the magnitude of BIS-induced block of *I*_K(M)_ taken from mHippoE-14 cells is indistinguishable from those described above in pituitary cells.

## 3. Discussion

BIS-induced suppression of *I*_K(M)_ presented herein was found to be attenuated by a further application of flupirtine, but not by isoproterenol or ractopamine, the agonist of β-adrenergic receptor. The present results are of particular importance, as they suggest that the inhibition of both *I*_K(M)_ and *I*_K(erg)_ caused by BIS is direct and appears to be unnecessarily linked to its binding to β-adrenergic receptor, although binding to β_1_-adrenergic receptor was previously reported to influence pituitary function [7,8,11,12,45]. However, distinguishable from the inhibitory effect of carvedilol, a non-selective blocker of β-adrenergic receptors, on the amplitude and inactivation kinetics *I*_K(DR)_ [13], BIS at a concentration greater than 10 µM slightly suppressed *I*_K(DR)_ amplitude with no discernible change in the inactivation time course of the current.

A previous report has demonstrated the ability of BIS to reverse the down-regulation in mRNA expression of small-conductance Ca^2+^-activated K^+^ channels [15]. In our study, neither continued presence of BIS, further addition of neither diazoxide, GMQ, nor 9-phenanthrol produced attenuating effects on its suppression of *I*_K(M)_. Diazoxide and 9-phenanthrol are known to stimulate ATP-sensitive and intermediate-conductance Ca^2+^-activated K^+^ channels, respectively, while 2-guanidine-4-methylquinazoline (GMQ) was reported to enhance the activity of large-conductance Ca^2+^-activated K^+^ channels [39,40]. In this scenario, the observed effect of BIS on whole-cell *I*_K(M)_ inherently in pituitary GH_3_ cells virtually is not involved in the suppression of either ATP-sensitive or Ca^2+^-activated K^+^ channels. The inhibitory effect on *I*_K(M)_ by BIS could be predominantly responsible for its decrease of macroscopic *I*_K(M)_ amplitude carried through K_M_ channels.

Block of *I*_K(M)_ caused by BIS is not instantaneous, but develops with time after the channels are opened upon rapid membrane depolarization, producing an apparent slowing in current activation. Deactivating current of *I*_K(M)_ in the presence of BIS showed a blunted peak and an increased decay that is consistent with the possibility that the closing (i.e., deactivating process) of channels was raised by unbinding of the BIS molecule. Moreover, as the falling phase upon return to hyperpolarizing potential (i.e., the down sloping ramp) was prolonged, the *I*_K(M)_ amplitudes were decreased exponentially. The magnitude of BIS-induced block on such deactivating currents was notably increased. The blocking site of this agent thus appears to be located within the channel pore only when the channel is open. KCNQ2/3 mRNA expression was detected in GH_3_ and mHippoE-14 cells; however, the present finding showed a lack of effect of BIS on single-channel conductance of K_M_ channels in GH_3_ cells, suggesting that the interaction of the channel with it seems to be secondary to alterations that are remote from the pore region of the channel.

Clinically relevant serum concentration of BIS was recently reported to range between 3.8 and 71.1 ng/mL (or 0.011 and 0.22 µM) [46]. These values are similar to the IC_50_ required for its suppression of *I*_K(M)_ presented herein. In this study, the IC_50_ value required for BIS-induced block of *I*_K(M)_ or *I*_K(erg)_ inherently in GH_3_ cells was 1.21 and 6.42 µM, respectively. This agent tends to be selective for *I*_K(M)_ over *I*_K(erg)_ or *I*_K(DR)_. There was also a depolarizing shift in the activation curve of K_M_ channels during exposure to BIS, strongly suggesting that this compound could alter the voltage dependence of channel activation accompanied by a decrease of free energy for K_M_-channel activation. During high frequency firing, the amplitude of *I*_K(M)_ was reduced and the sensitivity to BIS was attenuated. As the duration of the falling phase upon return from membrane potential of −10 to −50 mV increased, the magnitude of BIS-induced block of *I*_K(M)_ became raised. Taken together, the sensitivity of neurons or pituitary cells to BIS could depend not only on the BIS concentration achieved, but also on the pre-existing level of the resting potential, the discharge pattern or frequency, or their combinations. 

Our study showed that in pituitary cells, the presence of BIS does not appear to bind to β-adrenergic receptors exclusively, although the activity of these receptors might be constitutively active [47]. This compound was capable of suppressing *I*_K(M)_ and *I*_K(erg)_ directly and effectively. Under current-clamp recordings, BIS was effective at depolarizing the cell and raising the firing rate of spontaneous APs in GH_3_ cells. Subsequent addition of flupirtine, yet not ractopamine, could attenuate BIS-enhanced firing. Therefore, apart from its antagonistic effect on β-adrenergic receptors, the inhibition by BIS of these K^+^ currents may also conceivably contribute to its effect on the cellular function (e.g., stimulus-secretion coupling) in these cells. However, it also needs to be noted that the results presented here were derived from cell lines. Whether similar findings occurring in native cells still remains to be clarified.

Neither eugenolol nor eugenodilol was found to suppress *I*_K(M)_ in GH_3_ cells, although these newly designed compounds possess blocking actions on β-adrenergic receptors [48,49]. BIS or its structurally similar compounds may thus be a valuable tool for probing the structure and function of K_M_ or K_erg_ channels, because the pore region of the channel protein to which they bind is of particular relevance for an open-channel blockade. In addition to its antagonistic effect on β_1_-adrenergic receptors, the blockade of K_M_ and K_erg_ channels by BIS, together with an increase in the firing of spontaneous APs, may be responsible for its effects on pituitary or neuronal excitability. To what extent BIS-induced suppression of these K^+^ currents is responsible for its additional effectiveness with favorable metabolic profiles [2,50] has yet to be further delineated. Caution needs to be made in attributing its use to the selective blocking of β_1_-adrenergic receptors.

## 4. Materials and Methods

### 4.1. Chemicals and Solutions

(±)-Bisoprolol hemifumarate (BIS, zebeta^®^, (E)-but-2-enedioic acid;1-(propan-2-ylamino)-3-[4-(2-propan-2-yloxyethoxymethyl)phenoxy]propan-2-ol, C_13_H_31_NO_4_·½C_4_H_4_O_4_), 2-guanidine-4-methylquinazoline (GMQ), ML-213, PD-118057, and 9-phenanthrol were obtained from Tocris (Bristol, UK), while carvedilol, diazoxide, ethidium bromide, flupirtine, isoproterenol, ivabradine, linopirdine, ractopamine and tolbutamide were from Sigma-Aldrich (St. Louis, MO, USA). Pioglitazone was obtained from Takeda Pharmaceutical (Osaka, Japan), and parecoxib was from Pfizer Inc. (New York, NY, USA). Eugenolol and eugenodilol were gifts from Dr. Jwu-Lai Yeh, Department of Pharmacology, Kaohsiung Medical University, Kaohsiung City, Taiwan. Unless stated otherwise, the cell culture media, L-glutamine, horse serum and fetal calf serum were obtained from Invitrogen (Carlsbad, CA, USA). All other chemicals were commercially available and of analytical reagent grade. 

The composition of HEPES-buffered normal Tyrode’s solution was as follows (in mM): NaCl 136.5, KCl 5.4, CaCl_2_ 1.8, MgCl_2_ 0.53, glucose 5.5, and HEPES-NaOH buffer 5.5 (pH 7.4). To record membrane potential or *I*_K(M)_, the recording pipettes were filled with the solution (in mM); K-aspartate 130, KCl 20, KH_2_PO_4_ 1, MgCl_2_ 1, EGTA 0.1, Na_2_ATP 3, Na_2_GTP 0.1, and HEPES-KOH buffer 5 (pH 7.2). To record whole-cell *I*_K(M)_, high K^+^-bathing solution was composed of the following (in mM): KCl 145, MgCl_2_ 0.53, and HEPES-KOH 5 (pH 7.4). To measure the activity of single K_M_ channels, the pipette solution was composed of the following (in mM): NaCl 136.5, KCl 5.4, MgCl_2_ 0.53, and HEPES-NaOH buffer 5 (pH 7.4). All solutions were prepared using deionized water from a Milli-Q water purification system (APS Water Services, Inc., Van Nuys, CA, USA). The pipette solution and culture medium were commonly filtered on the day of use with Acrodisc^®^ syringe filter with 0.2 µm of Supor^®^ membrane (Pall Corp., Port Washington, NY, USA).

### 4.2. Cell Preparations

GH_3_ pituitary tumor cells, obtained from the Bioresources Collection and Research Center ([BCRC-60015]; Hsinchu, Taiwan; originally derived from ATCC [CCL-82.1^TM^], were maintained in Ham’s F-12 medium supplemented with 15% horse serum (*v*/*v*), 2.5% fetal calf serum (*v*/*v*), and 2 mM L-glutamine in a humidified environment of 5% CO_2_/95% air. Rat pituitary cells (#R1220) were purchased from ScienCell Research Laboratories, Inc. (Carlsbad, CA, USA). These cells reported to express the receptors of gonadotropin releasing hormone (GnRH) were isolated from neonate day-8 CD^®^ rats and cryopreserved in primary cultures with further purification and expansion (https://www.sciencellonline.com/products-services/primary-cells/animal/cell-types/pituitary cells/rat-pituitary-cells.html) [51,52]. Cells were routinely grown in Epithelial Cell Medium (Cat #4101; ScienCell). 

Embryonic mouse hippocampal cell line (mHippoE-14, CLU198) was obtained from Cedarlane CELLutions Biosystems, Inc. (Burlington, ON, Canada) [39,53,54]. Cells were maintained in Dulbecco’s modified Eagle’s medium supplemented with 10% fetal bovine serum (*v*/*v*) and 2 mM L-glutamine. The culture medium was changed every two to three days, and cells underwent passaged when they reached confluence. The experiments were commonly performed 5 or 6 days after cells had been cultured (60–80% confluence).

### 4.3. RNA Isolation and Reverse Transcriptase-Polymerase Chain Reaction (RT-PCR)

To detect the expression of rat KCNQ2 and KCNQ3 mRNAs in different types of cells including GH_3_, R1220 cells, a semiquantitative RT-PCR assay was performed. Total RNA samples were extracted from mHippoE-14 cells with TRIzol reagent (Invitrogen) and reverse-transcribed into complementary DNA using Superscript II reverse-transcriptase (Invitrogen). The sequences of forward and reverse oligonucleotide primers used for KCNQ2 were 5′-CCCTGAAAGTCCAAGAGCAG-3′ and 5′-AGGCCCCATAGGTTTGAGTT-3′, respectively, while those for KCNQ3 were 5′-GTGGCTTCAGCATCTCACAA-3′ and 5′-CTTGTTGGAAGGGGTCCATA-3′, respectively. Amplication of KCNQ2 or KCNQ3 was made using PCR SuperMix from Invitrogen under the following conditions: 35 cycles composed of 30 sec denaturation at 95 °C. 30 sec primer annealing at 62 °C, 1 min extension at 72 °C, and followed by 72 °C for the final extension for 2 min. PCR products were analyzed on 1.5% (*v*/*v*) agarose gel containing ethidium bromide and then visualized under ultraviolet light. Optical densities of DNA bands were scanned and quantified by AlphaImager 2200 (ProteinSimple; Santa Clara, CA, USA).

### 4.4. Electrophysiological Measurements

Immediately prior to the experiment, cells were dissociated and an aliquot of cell suspension was transferred to a custom-made recording chamber mounted on the stage of a DM-IL inverted microscope (Leica, Wetzlar, Germany). There were immersed at room temperature (20–25 °C) in HEPES-buffered normal Tyrode’s solution, the composition of which is described above. The patch electrodes were fabricated from borosilicate glass capillaries (No. 34500; Kimble Products, Vineland, NJ, USA) on a Narishige PP-83 puller (Narishige, Tokyo, Japan) or a P-97 Flaming/Brown puller (Sutter, Novato, CA, USA), and electrode tips were fire-polished with MF-83 microforge (Narishige). Their resistances in standard pipette and bathing solutions ranged from 3 to 5 MΩ. Recordings of membrane potential or ion currents were measured in the whole-cell or cell-attached mode of the patch-clamp technique with an RK-400 patch-clamp amplifier (Bio-Logic, Claix, France) [42]. The liquid junction potentials were corrected shortly before seal formation was established.

### 4.5. Data Recordings

The data comprising both potential and current traces were stored online in an Acer SPIN−5 touchscreen laptop computer (SP513-52N-55WE; Taipei, Taiwan) at 10 kHz equipped with Digidata 1440A interface (Molecular Devices, Inc., Sunnyvale, CA, USA), which was used for the analog-to-digital/digital-to-analog conversion. During the recordings, the latter device was controlled by pCLAMP 10.7 software (Molecular Devices) run under Windows 10 (Redmond, WA, USA), and the signals were simultaneously monitored on LCD monitor through a USB type-C connection. Current signals were low-pass filtered at 2 kHz with a FL-4 four-pole Bessel filter (Dagan, Minneapolis, MN, USA) to minimize background noise. Through digital-to-analog conversion, various pCLAMP-generated voltage-clamp profiles with either rectangular or ramp waveforms were applied to determine the current-voltage (*I-V*) relationship of *I*_K(M)_, *I*_K(erg)_ or *I*_K(DR)_. As high-frequency stimuli were necessarily applied to the cell, an Astro-med Grass S88X dual output pulse stimulator (Grass Technologies, West Warwick, RI, USA) was used. After the data were digitally collected, we later analyzed them using different analytical tools that include LabChart 7.0 program (AD Instruments; Gerin, Tainan, Taiwan), OriginPro 2016 (Microcal, Northampton, MA, USA), or custom-made macros built under Microsoft Excel^®^ 2013 (Redmond).

### 4.6. Data Analyses

To determine percentage inhibition of BIS on *I*_K(M)_ or *I*_K(erg)_, we compared the current amplitudes during cell exposure to different BIS concentrations. To record *I*_K(M)_, we bathed the cells in high-K^+^, Ca^2+^-free solution, and depolarizing step from −50 to −10 mV was applied, while, to measure *I*_K(erg)_, the examined cell was hyperpolarized from −10 to −100 mV. The concentration-response data for inhibition of *I*_K(M)_ or *I*_K(erg)_ were least-squares fitted by a modified Hill function. That is,

$$\mathrm{Percentage}\;\mathrm{inhibiton}\;=\;\frac{E_{max}\times {\left[BIS\right]}^{n_{H}}}{IC_{50}^{n_{H}}+{\left[BIS\right]}^{n_{H}}},$$

where [BIS] represents the BIS concentration; IC_50_ and n_H_ are the concentration required for 50% inhibition and the Hill coefficient, respectively; and E_max_ is the maximal inhibition of *I*_K(M)_ or *I*_K(erg)_ induced by this drug.

### 4.7. Analyses of Single M-Type K^+^ (K_M_) Channels

Single K_M_-channel currents recorded from GH_3_ cells or mHippoE-14 neurons were analyzed using pCLAMP 10.7. We determined single-channel amplitudes taken with or without the BIS addition by fitting Gaussian distributions to the amplitude histograms of the closed and open states. The probabilities of K_M_ channel that would be open were defined as *N*·*P*_O_, which is estimated using the following expression:

$$N\cdot P_{O}\;=\;\frac{A_{1}+2A_{2}+3A_{3}+\ldots +nA_{n}}{A_{0}+A_{1}+A_{2}+\ldots +A_{n}}$$

where *N* is the number of active channels in the patch, A_0_ the area under the curve of an all points histogram corresponding to the closed state, and A_1_…A_n_ the histogram area that indicate the level of the distinct open state for 1 to *n* channels in the patch examined. The single-channel conductance of K_M_ channels was calculated using a linear regression with averaged values of single-channel amplitudes measured at different levels of membrane potentials relative to the bath. Open lifetime distributions of K_M_ channels taken with or without BIS addition were least-squares fitted with logarithmically scaled bin width.

To determine voltage-dependence of the inhibitory effect of BIS on the activity of K_M_ channels, the patch with or without BIS addition was held at different membrane potentials. The activation curve of K_M_-channel openings taken with or without addition of BIS (3 μM) was fitted by the Boltzmann equation:

$$\mathrm{Relative}\;\mathrm{open}\;\mathrm{probability}\;=\;\frac{P_{max}}{1+e^{\left[\frac{-\left(V-V_{1/2}\right)\mathrm{qF}}{\mathrm{RT}}\right]}}\;,$$

where P_max_ is the maximal probability of K_M_-channel openings in the control (i.e., in the absence of BIS) maintained at +30 mV relative to the bath, V_1/2_ the voltage at which half-maximal activation of K_M_ channels occurs, q the apparent gating charge, F Faraday’s constant, R the universal gas constant, T absolution temperature, and F/RT = 0.04 mV^−1^.

### 4.8. Statistical Analyses

The linear or nonlinear (e.g., sigmoidal or exponential curves) least-squares fitting routines were appropriately estimated using either the Solver add-in bundled with Microsoft Excel^TM^ 2013 (Redmond) or OriginPro 2016 (OriginLab, Northampton, MA, USA). The values appearing in this study are provided as the mean values ± standard error of mean (SEM) with sample sizes (*n*) indicating the number of cells from which the experimental data were taken, and error bars are plotted as SEM. In this study, we intended to make assertions about the variability of means that could be collected from a random cohort derived from the population concerned. For this reason, the standard error could be more appropriate than the standard deviation. The paired or unpaired Student’s *t*-test, or one-way analysis of variance followed by post-hoc Fisher’s least-significance difference test for multiple-group comparisons, were implemented for the statistical evaluation of differences among means. We used non-parametric Kruskal-Wallis test, as the assumption of normality underlying ANOVA was violated. Statistical analyses were performed using IBM SPSS^®^ version 20.0 (IBM Corp., Armonk, NY, USA). A difference with a *p* value < 0.05 was considered statistically significant, unless otherwise stated.

## Abbreviations

AP	action potential
BIS	bisoprolol
*erg*	ether-à-go-go-related gene
GMQ	2-guanidine-4-methylquinazoline
*I-V*	current versus voltage
IC50	the concentration required for 50% inhibition
*IK(DR)*	delayed-rectifier K^+^ current
*IK(erg)*	erg-mediated K^+^ current
*IK(M)*	M-type K^+^ current
Kerg	erg-mediated K^+^ channel
KM channel	M-type K^+^ channel
SEM	standard error of mean
τact	activation time constant
τdeact	deactivation time constant.
